# Idiopathic multifocal choroiditis (MFC): aggressive and prolonged therapy with multiple immunosuppressive agents is needed to halt the progression of active disease. An offbeat review and a case series

**DOI:** 10.1186/s12348-021-00278-8

**Published:** 2022-01-10

**Authors:** Ioannis Papasavvas, Piergiorgio Neri, Alessandro Mantovani, Carl P. Herbort

**Affiliations:** 1 Retinal and Inflammatory Eye Diseases, Centre for Ophthalmic Specialized Care (COS), Rue Charles-Monnard 6, 1003 Lausanne, Switzerland; 2 The Eye Institute, Cleveland Clinic Abu Dhabi, Abu Dhabi, United Arab Emirates; 3 Cleveland Lerner College of Medicine, Case Western University, Cleveland, OH USA; 4 Khalifa University, Abu Dhabi, United Arab Emirates; 5 Department of Ophthalmology, Valduce Hospital, Como, Italy

**Keywords:** Multifocal choroiditis, FA, ICGA, Primary inflammatory choriocapillaropathies

## Abstract

**Background and purpose:**

Idiopathic multifocal choroiditis (MFC) is part of the group of choriocapillaritis entities. The clinical definition of the disease has evolved with time. The aim of this article was to undertake a review on MFC, on its present-day appraisal and nomenclature and we also report a series of patients with emphasis on the clinical presentation and the importance of vigorous immunosuppressive management.

**Methods:**

A review of the literature and a retrospective case series study which was performed in the Centre for Ophthalmic Specialised care (COS), Lausanne, Switzerland. Patients diagnosed from 1994 to 2020 with idiopathic multifocal choroiditis (MFC) treated with multiple immunosuppressants were included. Exclusion criteria were insufficient follow up and cases not treated with vigorous immunosuppressive therapy. Imaging analysis included spectral domain optical coherence tomography (SD-OCT) / enhanced depth imaging OCT (EDI-OCT), OCT angiography (OCT-A). Fluorescein and Indocyanine angiography (FA, ICGA) before and after the instauration of treatment. Best corrected visual acuity (BCVA), intraocular pressure (IOP), routine ocular examination, laser flare photometry (LFP) were performed at presentation and follow-up. Immunosuppression comprised at minimum two among the following agents: prednisone, cyclosporine, azathioprine, mycophenolic acid or infliximab. Mean duration of therapy was calculated.

**Results:**

26 (52 eyes) of 2102 new patients (1.24%) were diagnosed with MFC. 25 (96%) patients were female and 1 (4%) was male. 43/52 (82%) eyes were myopic with a mean dioptre of − 5.87 ± 2.94, six (12%) eyes were hypermetropic with mean dioptres 2.0 ± 2.68 and three (6%) were emmetropic. 14/52 (27%) eyes had at least 1 anti-VEGF injection because of choroidal neovascularisation (CNVs), 1 eye had a phototherapy laser and 37/52 (71%) had no complication of CNVs during the follow-up. 5/26 (19%) fulfilled the inclusion criteria for our study. Mean age was 26.4 ± 9.3 years. Snellen best corrected visual acuity (BCVA) at presentation was 0.955+/-0.26. Mean follow up was 84+/-55 months. LFP at presentation was 6.34 ± 2.94 ph/ms. None of four patients with prolonged treatment and prolonged follow-up showed disease activity. One patient still under therapy after 4 months’ follow-up still showed an active neovascular membrane.

**Conclusion:**

Treatment with multiple immunosuppressive agents was shown to stop the progression of the disease.

## Introduction/historical aspects

Idiopathic Multifocal Choroiditis (MFC) is a choriocapillaritis also called primary inflammatory choriocapillaropathy (PICCP) not linked to a known infectious agent. It predominantly affects healthy myopic white women and is characterised by uni or bilateral chorioretinal lesions which have often a recurrent course with subclinical novel recurrent lesions identified by indocyanine green angiography (ICGA), coexistent with older scarred chorioretinal lesions and frequently complicated by choroidal neovessels (CNVs).

The term of” multifocal choroiditis” with a description corresponding to the present appraisal of the disease was used by Krill et al. in 1969 who named the disease multifocal inner choroiditis [1].

Thereafter the condition was overlapping or confused in different reports with ocular histoplasmosis later called presumed ocular histoplasmosis syndrome (POHS) [2].

In 1973, Nozik and Dorsch described an entity which they called multifocal uveitis and panuveitis [3]. In 1984 Dreyer and Gass published a report entitled “multifocal choroiditis and panuveitis, a syndrome that mimics ocular histoplasmosis syndrome”, reporting 28 additional cases [4]. The panuveitis part in most cases is very minimal and mostly limited to cells in the posterior vitreous and, nowadays, should not be considered as a disease defining sign. The first reports on the utility of ICGA in MFC were published in 1996–97 [5, 6]. The extreme sensitivity of ICGA to follow and monitor active disease was established thereafter [7]. ICGA made it clear that MFC typically affects primarily the choriocapillaris and should therefore be classified in the group of PICCPs [8].

The more severe course of MFC when compared to multiple evanescent white dot syndrome (MEWDS) or acute posterior multifocal placoid pigment epitheliopathy/acute multifocal choriocapillaritis (APMPPE/AMIC) was made clear rapidly as well as its propensity to develop CNVs [9]. In 1984 was published the first report on punctate inner choroidopathy (PIC) difficult to distinguish from MFC [10]. In 1998, a histopathological report on choroidal lesions in MFC showed inflammatory involvement of the choroidal stroma in addition to choriocapillaris involvement [11]. This extension of inflammation to deeper pre-choriocapillaris vessels might be an explanation both for being in the more severe disease spectrum of PICCPs and for the higher frequency of CNVs [11, 12]. Even inner retinal vessels showed scarce perivascular inflammatory infiltration [11].

The boundaries and nosological characteristics of MFC are less well determined and the entity is more heterogeneous than MEWDS or APMPPE/AMIC. When seen by the clinician most of the cases already show chorioretinal scars from preceding silent episodes before the disease becomes symptomatic during a recurrence. The terminology of the different forms that constitute idiopathic multifocal choroiditis will be discussed in the next section. The characteristics of all the subtypes of multifocal choroiditis are the numerous small randomly distributed chorioretinal scars and the recurrent behaviour of the disease as well as the propensity to develop secondary CNVs, which, as said before, is much more frequent than in all other PICCPs. Multifocal choroiditis occurs in the same age groups as other PICCPs, namely in young to middle aged adults with women being predominantly affected [9]. Lesions tend to leave scars, are not spontaneously reversible but seem to respond to corticosteroid therapy and/or non-steroidal immunosuppressive agents which is a recommended treatment.

The aim in this article was to apply pioneering pragmatism in the approach and definition of a disease with many different confusing denominations and present our experience in the appraisal and management of the disease.

## Epidemiological aspects and nomenclature

### Epidemiology

It is very difficult to establish epidemiological data for MFC as the disease comprises entities that were classified under different terms in the past, which were grouped under the terminology of idiopathic multifocal choroiditis only recently [13]. However, even recent studies still report MFC and PIC, and others as separate entities. When performing a literature search containing the terms of “idiopathic multifocal choroiditis” and “epidemiology” no publication could be found in the PubMed databank. One of the most significant publication reported a series of 41 patients with a mean age of 38.4 years, predominantly women (70,7%) and a bilaterality rate of 51.2% [14].

In a study published in 2015, the frequency of diagnosis of MFC in different US uveitis centres was 1.8%, 6%, 6.5% and 4.9% [15]. In our centre, from 1994 to 2020, MFC was diagnosed in 26/2102 new uveitis patients amounting to a frequency of 1.24% of uveitis diagnoses. Although epidemiological information is scarce and difficult to obtain, it is obvious that MFC is a rare uveitis.

#### Nomenclature considerations (Table 1)

Idiopathic multifocal choroiditis (MFC), the eponym that should be used today for the disease, is a non-infectious primary inflammatory choriocapillaropathy (PICCP) for which no infectious agent has been identified. It should be distinguished from presumed ocular histoplasmosis syndrome (POHS) which is an infectious entity caused by histoplasma capsulatum that used to be called ocular histoplasmosis syndrome [26]. The term of presumed was quickly added because, despite a positive histoplasmin skin test, as the presence of the infectious agent has not been specifically identified in the eye [16, 27, 28]. POHS can be applied to characteristic ocular findings with a positive histoplasmin skin test and/or patients living in an endemic area.

**Table 1:** Chronology of significant publications corresponding to today’s (idiopathic) multifocal choroiditis and those that influenced the eponym

Year	Title	Authors	References
1959	*The probable role of benign histoplasmosis in the etiology of granulomatous uveitis* (1)	Woods AC & Wahlen HE	Trans Am Oph Soc 1959; 57:318 [16**]**
1969	Multifocal inner choroiditis (2)	Krill AE et al.	Trans Am Acad Opht Otolaryn 1969;73:222–45 [1]
1970	*Choroidal neovascularization in multifocal (presumed histoplasmin) choroiditis* (1)	Krill AE & Archer D	Arch Opht 1970; 84:595–604 [2]
1975	Multifocal choroiditis	Archer DB & Maguire CJ	Trans Opht Soc UK 1975; 95:184–91 [17]
1975	Diagnosis and treatment of macular lesions in multifocal choroiditis (presumed histoplasmosis) (3)	Notting JG & Deutman AF	Klin Monbl Aug. 1975 May;166(5):629–36 [18]
1976	De novo lesions in presumed histoplasmosis-**like (POHS)** syndrome (4)	Miller SA et al.	Br J Opht 1976; 60:700–12 [19]
1984	Multifocal choroiditis and panuveitis. A syndrome that mimics ocular histoplasmosis	Dreyer RF & Gass DJ	Arch Opht 1984; 102; 1776–84 [4]
1985	Inflammatory pseudohistoplasmosis (POHS) (4)	Deutsch TA & Tessler HH	Ann Opht 1985; 17:461–5 [20]
1986	Multifocal inner choroiditis: pseudohistoplasmosis. The European form of the presumed American histoplasmosis (4)	Saraux H et al	J Fr Ophtalmol186; 9:645–51 [21]
1986	Recurrent multifocal choroiditis	Morgan CM & Shatz H	Ophthalmology 1986; 93:1138–47 [22]
1990	Multifocal choroiditis (5)	Joondeph BC & Tessler HH	Int Ophthalmol Clin. Fall 1990; 30:286–90 [23]
1995	Fundal white dots: the spectrum of a similar pathological process (6) **	Ben Ezra D & Forrester JV	Br J Ophthalmol 1995; 79:856–60 [24]
1997	**Indocyanine green angiography of multifocal choroiditis**	Slakter et al.	Opht 1997; 104:1813–9 [6]
1998	Multifocal choroiditis: clinicopathologic consideration	Dunlop AA et al.	Arch Ophthalmol 1998; 116:801–3 [11]
1998	Histoplasmosis-like choroiditis in a nonendemic area: the north-western United Sates (5)	Watzke RC e al.	Retina 1998; 18:204–12 [25]
2013	**Idiopathic multifocal choroiditis: a comment on present and past nomenclature**	Essex RW et al.	Retina 2013; 33:1–4 [13]
	(1) *Among first articles implicating histoplasmosis in POHS, in italics so to distinguish it from MFC*		
	(2) Can probably be considered as the first article on MFC		
	(3) Uses term of histoplasmosis but histoplasmin skin test negative cases that correspond to MFC		
	(4) Apparition of terms of pseudohistoplasmosis, pseudo-POHS or histoplasmosis-like corresponding to MFC cases		
	(5) Comprehensive summary including historical publications on MFC		
	(6) ** Unfortunate terminology which included MFC within a group of other unrelated entities		
	**In bold, significant progress in the appraisal and nomenclature of MFC**		
	*In italics publications on POHS cases, distinct from MFC*		

POHS or even ocular histoplasmosis have unfortunately been used in non-endemic areas and/or in histoplasmin negative patients [18, 21, 29] and was sometimes called pseudo-POHS [25, 30]. This is at the origin of part of the confusion in the nomenclature of MFC.

MFC was named by a record number of terminologies, including multifocal inner choroiditis being probably the first description, by Krill et al. in 1969 [1], punctate inner choroidopathy (PIC) [10], POHS (in non-endemic areas or with a negative histoplasmin skin test) [25] or pseudo-POHS [31]. Other alternative names given to the disease in the past were recurrent multifocal choroiditis [22], multifocal choroidopathy or disseminated inner choroiditis [21].

Some reports called the disease multifocal choroiditis with panuveitis [3, 4]. Although some cases may present panuveitis, these unusual forms are seemingly sufficiently rare that the term of panuveitis does not characterise MFC and cannot be considered as a disease defining sign. Indeed, the fact that a study was able to publish 41 patients with MFC without panuveitis supports this position [14]. In our 26 MFC patients none presented panuveitis.

In 2013 a salutary editorial was published to group the differently termed conditions under the eponym of idiopathic multifocal choroiditis [13]. While a very large series of MFC without panuveitis is available [14] and a very clear and comprehensive definition is available [13], it is unfortunate that a recent classification continued to call the disease “multifocal choroiditis with panuveitis”, instead of using the consecrated term of idiopathic multifocal choroiditis, panuveitis not being the hallmark of the condition [32].

Anecdotally, the confusion in terminology became apparent to one of the authors back in 2003, when a fellow from a neighbouring country was sent to analyse ICG angiographies of European patients with so-called POHS. Looking at the angiographies the cases corresponded to what was considered then by us and is now commonly called idiopathic multifocal choroiditis.

**Fig. 1:**
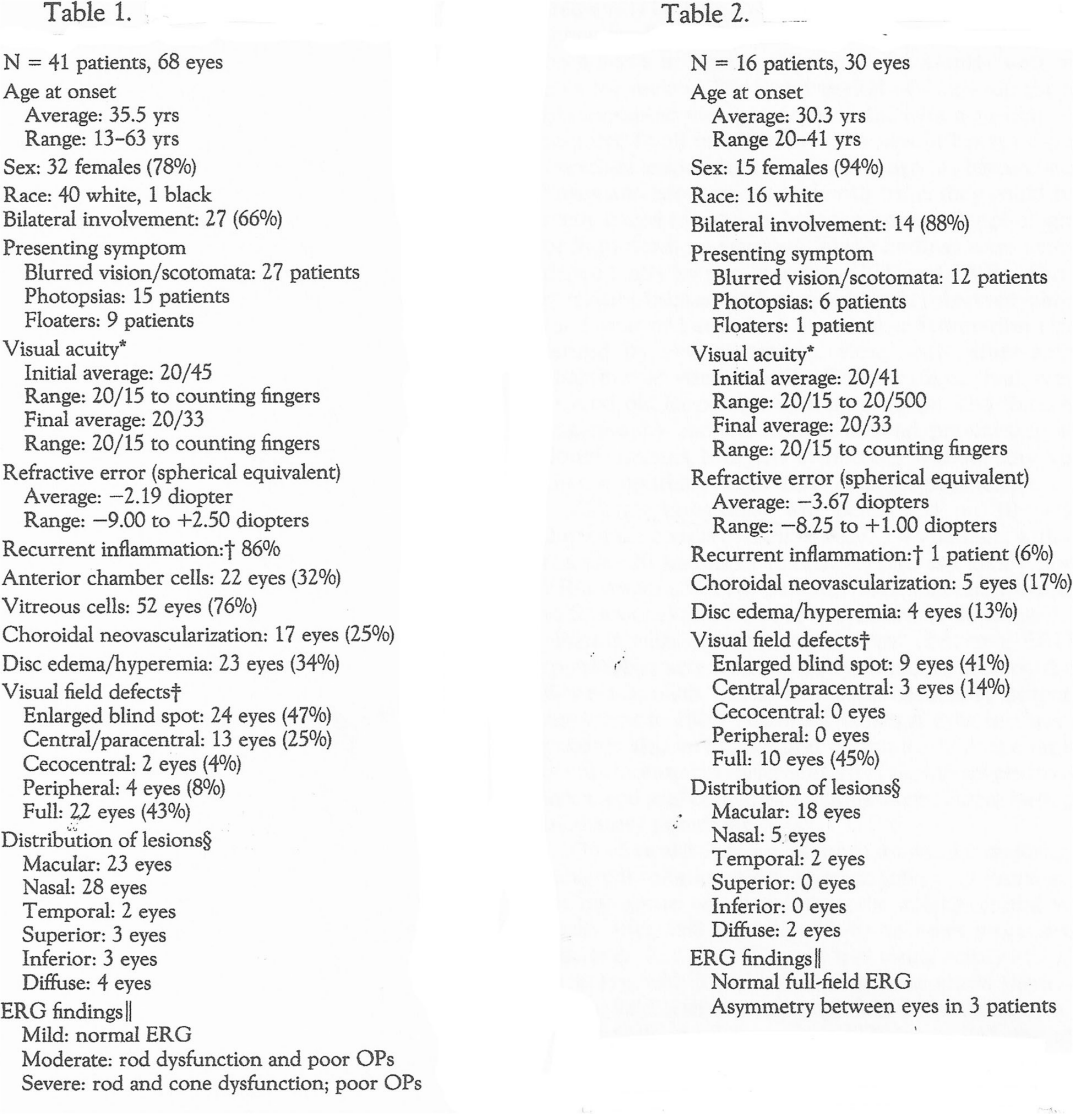
MFC and PIC are undistinguishable. Two tables defining MFC and PIC for which the titles have been hidden. The two “entities” cannot be distinguished and statistical differences among items do not appear to be significant. This is one more argument that the two “sub-entities” are one and the same disease [33].

Up to date, despite the comprehensive grouping of sub-entities into MFC, there are still reports that distinguish PIC from MFC. When looking at the definition of one and the other of these two” entities” on the tables of an extensive work on sub-entities of MFC, it appears impossible to distinguish the two forms when the titles of the tables are hidden (Fig. 1) [33].

Table 1. Shows the impact of presumed ocular histoplasmosis syndrome (POHS) on the appraisal of MFC, by slowing the progressive distinction of the specific MFC entity and the confusion on the terminology in the early years such as the European publication by Notting and Deutman in 1975 [30], speaking of presumed histoplasmosis in patients outside endemic areas and with negative histoplasmin skin tests. (Non-exhaustive listing performed on available articles and left to the fair appreciation and careful choice of the authors).

## Pathology and clinicopathology

The trigger for MFC is still unknown. Like for other PICCPs, it might be an unidentified infectious agent, such as viruses, as many patients in this group can present flu-like symptoms before the onset of the ocular disease. Several cases of multifocal choroiditis with a similar presentation to the idiopathic form have been linked to infectious agents, including herpes zoster virus [34, 35], Epstein-Barr virus [36–38], West Nile virus [39] and Zika virus [40]. The clinicopathology/disease process, however, seems to be immune-mediated non-perfusion of the choriocapillaris, as in other entities of the group, including MEWDS, APMPPE/AMIC and serpiginous choroiditis. This is very well shown by ICGA that characterises the process, identifying choriocapillaris non-perfusion in new developing lesions. Additional arguments for an immune mediated process are (1) several reports indicating that multifocal choroiditis developed after different types of vaccinations [41–43], and (2) the fact that the disease responds well to immunosuppression. Choriocapillaris non-perfusion is causing secondary ischaemia of the outer retina damaging photoreceptor outer segments well identified by spectral domain optical coherence tomography (SD-OCT) and by blue-light fundus autofluorescence (BL-FAF) showing hyperautofluorescence in the affected areas due to the loss of the photoreceptor screen to retinal pigment epithelium autofluorescence.

Anatomopathological reports are understandably scarce. In a case of clinicopathological correlation, the clinical lesions corresponded to non-granulomatous perivascular choroidal infiltrates, consisting mainly of B lymphocytes supporting the fact that choroidal vessels are at the origin of the clinicopathology of MFC [11]. The fact that lesions tend to evolve to chorioretinal scars in MFC suggests that larger pre-choriocapillaris vessels are involved. Another report on excised CNVs from MFC also showed infiltration by B lymphocytes [44]. The different grades of severity of PICCPs might be explained by the choroidal vessel calibre involved and the severity of the process. (Table 2).

**Table 2:**
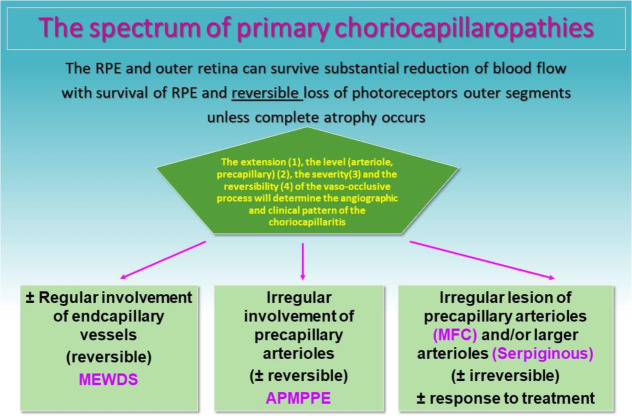
Schematic classification of PICCPs according to suspected location of vaso-occlusive event. (Adapted from “Diagnostics (Basel). 2021 May 24;11 (6):939. doi: 10.3390/diagnostics11060939.)

The clinicopathology appears to be identical in the two main sub-entities, MFC and PIC, described separately before the unifying terminology of idiopathic multifocal choroiditis was adopted. Distinguishing and comparing MFC and PIC has often been attempted in the past but corresponded to a vain exercise because they should be considered as part of the same disease, as is apparent on Fig. 1. Moreover, MFC and PIC were shown to have similar genetic associations another argument for a unique disease [45].

## Clinical presentation

### Symptoms

The symptoms that connect multifocal choroiditis to all other PICCPs are the photopsias and subjective scotomas. Photopsia is usually much more disturbing for the MFC patient than for other PICCPs and their duration is protracted, being present also when there is no clinical evidence of reactivation of the disease. The patients also report more frequent subjective scotomas. Multifocal choroiditis can be bilateral with involvement being usually asymmetric or sequential, first detected in one eye and months or even years later in the fellow eye. When it is unilateral it has often been included in the subtype of PIC, a terminology that should now be abandoned. Recurrences are usual and can be documented with fundoscopy and ICGA [23].

### Clinical signs

Visual acuity impairment is variable and depends on the area of involvement and the rapidity of immunosuppressive treatment implementation.

Only slight non granulomatous anterior segment inflammation can be seen. Therefore, if anterior granulomatous uveitis is present a specific diagnosis, such as sarcoidosis, syphilis or tuberculosis has to be looked for. Cells in the posterior vitreous can be found most of the time when the disease is active but can be absent in quiet disease. In none of our patients did we note what can be termed panuveitis, except for slight signs of posterior vitreous cells. Typically, when present, aqueous flare, measured by laser flare photometry (LFP) is below 20 ph/ms (normal values 3–5 ph/ms).

**Fig. 2:**
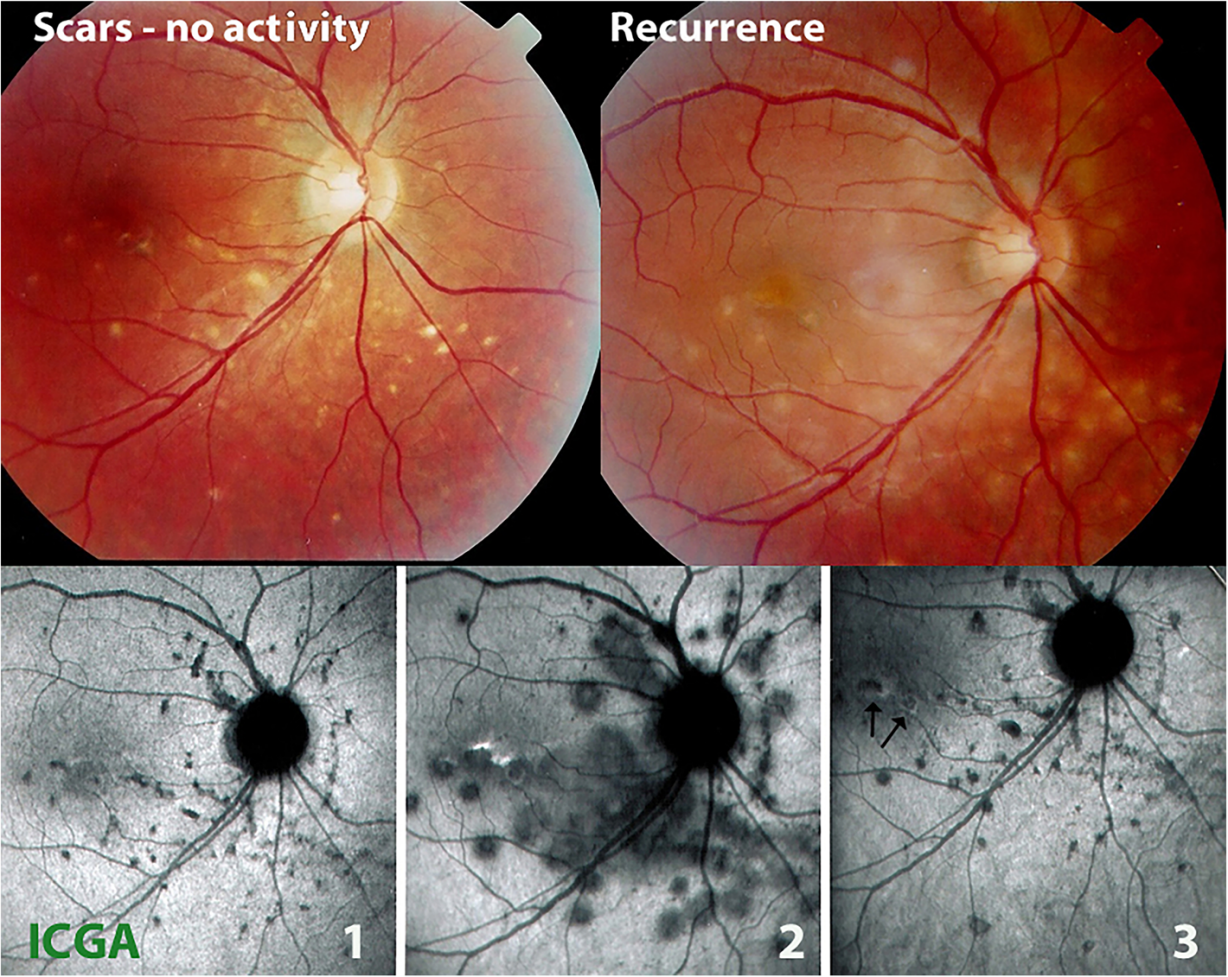
Multifocal choroiditis (MFC) Fundus pictures (top two images): on the left picture, multiple yellow punched-out foci typically seen in MFC (inactive stage); on the right picture, additional new more fluffy lesions during recurrence. ICGA pictures (bottom), during inactive cicatricial stage (1), during reactivation (2), and after treatment (3); almost no additional scars. (Partially reprinted from Diagnostics (Basel). 2021 May 24;11 (6):939. doi:10.3390/diagnostics11060939)

On fundus examination, the typical lesions are small punched-out randomly distributed choroidal mostly atrophic yellow-white foci with pigment spots that sometimes can become adjacent to each other and form a ribbon of pearls (Fig. 2). These lesions involve predominantly the posterior pole with a size around 70–100 μm, but peripheral lesions can also be seen, reported by several authors as peripheral linear streaks or Schlaegel lines [30, 46, 47]. In the active phases of disease new lesions are not always visible and can be very discreet on FA, whereas ICGA is the most sensitive method to detect new lesions [6, 7, 48]. Fundus lesions may be of different ages, signs of different episodes of recurrence. One particular feature of multifocal choroiditis is the high proportion of CNVs complicating the disease [12, 49, 50].

## Imaging and investigations

### Indocyanine green angiography (ICGA)

ICGA represented a breakthrough in understanding the clinicopathological process of MFC as well as its appraisal and management [6]. It allowed to detect early signs of incipient episodes showing choriocapillaris hypo or non-perfusion.

**Fig. 3:**
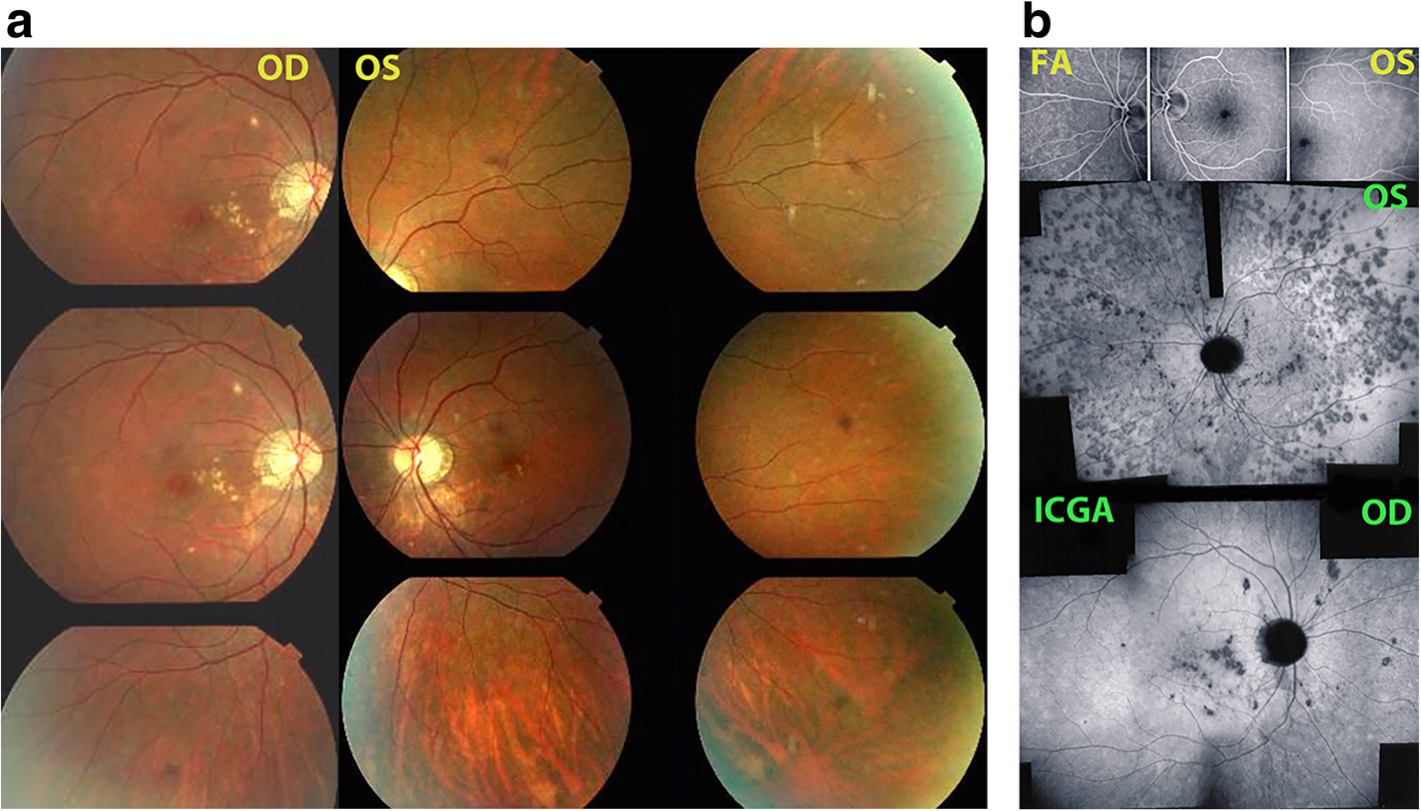
Multifocal choroiditis (MFC) Fundus pictures ODS. Typical chorioretinal scars OD in inactive stage. OS minimal number of scars in an eye with photopsias and subjective scotomas with widespread occult choriocapillaritis (see Fig. 3b). b. Multifocal choroiditis (MFC) Occult choriocapillaritis. ICGA and FA in same patient as Fig. 3a. OS: vast areas of ICGA hypofluorescence indicating choriocapillaris hypo or non-perfusion. These zones indicate that in some cases the inflammatory process is involving vast areas of choriocapillaris non-perfusion that can be a trigger for the development of CNVs. In OD ICGA only shows inactive hypofluorescent scars without occult activity. FA (3 top frames) shows no more than slight late hyperfluorescence (middle and right frames)

**Fig. 4:**
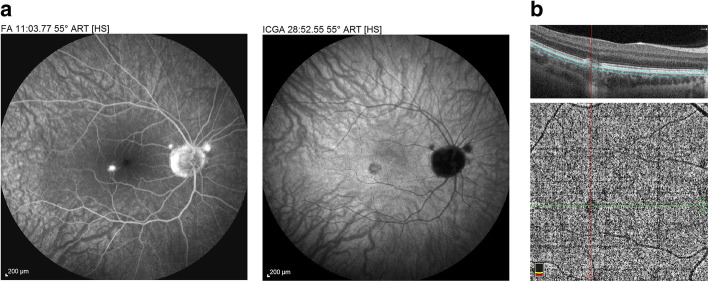
Multifocal choroiditis (MFC): CNVs. Small parafoveal CNV brightly hyperfluorescent on FA (left frame) and also hyperfluorescent on ICGA (right frame). On the other hand, the two paramacular atrophic scars are hyperfluorescent on FA (left frame) but are hypofluorescent on ICGA (right frame). b. Multifocal choroiditis (MFC) - CNVs. Shown by OCTA (same patient as Fig. 4a)

The first set of signs identifies old scarred chorioretinal lesions and consists of hypofluorescent areas persisting up to the late angiographic phase, distributed at random in the fundus, corresponding to late hyperfluorescence on fluorescein angiography. This constellation is typical for chorioretinal atrophy from scars of previous inflammatory episodes seen on fundus examination [Figs. 3a & b]. The second set of signs can be seen in addition to the previously described signs when choroiditis recurs or can be seen in their absence when it is the first episode of MFC. The signs consist of hypofluorescent areas, either silent on fluorescein angiography or slightly hyperfluorescent in the late angiographic phase and usually not visible on fundus examination, representing areas of new inflammatory involvement /choriocapillaris non-perfusion (Figs. 3a & b). As in MEWDS, some cases may present peripapillary hypofluorescence translating functionally into an enlarged blind spot [33, 51] (Fig. 2, middle bottom picture) The latter set of signs responds to systemic corticosteroids with or without immunosuppressants and can regress completely if therapy is started early. In a substantial proportion of cases the extent of ICGA hypofluorescence reflecting choriocapillaris hypoperfusion or non-perfusion is far more widespread than visible lesions let suspect, showing widespread areas of late hypofluorescence with absolutely no signs visible on fundus examination or on fluorescein angiography **(**Figs. 3a & b). It is probably such undetected chronic choriocapillaritis that explains the high proportion of CNVs, as inflammation can be the trigger of CNVs [12]. Indeed, ICGA is useful in distinguishing inflammatory lesions from CNVs, as the latter are hyperfluorescent (Figs. 4a & b). Moreover ICGA, together with OCT angiography (OCT-A) can monitor the evolution of CNVs after anti-vascular endothelial growth factor (anti-VEGF) treatment.

Furthermore, ICGA was shown to be a precious modality to distinguish between POHS that presented hyperfluorescent spots and MFC characterised only by hypofluorescent dots [52].

### Fluorescein angiography (FA)

FA is marginally useful in the active inflammatory phase of MFC, as indicated by Slakter et al. [6]. It shows mainly signs of chorioretinal scarred lesions associating window effects (late sclera hyperfluorescence due to staining) to masking effects where there is pigment clumping. In the active phase FA may show faint late hyperfluorescence (Fig. 3b, top 3 frames) in areas corresponding to ICGA hypofluorescent dark dots, the ICGA expression of new lesions. In case of severe hypoperfusion of the choriocapillaris, bright late hyperfluorescence (retinal and subretinal staining and even pooling) can occur as for APMPPE/AMIC (see hereunder). The use of fluorescein angiography is however of little contribution to assess and follow active lesions, as FA angiographic signs are often absent or faint in new areas of inflammatory involvement that are, in contrast, clearly shown by ICGA (Fig. 3b). In case of late hyperfluorescence, FA responds in a delayed fashion following the introduction of corticosteroid/immunosuppressive treatment, accounting less precisely for the resolution of new lesions than ICGA. In case of CNV, FA shows the classical signs of leakage and late hyperfluorescence.

### Optical coherence tomography (OCT)

**Fig. 5:**
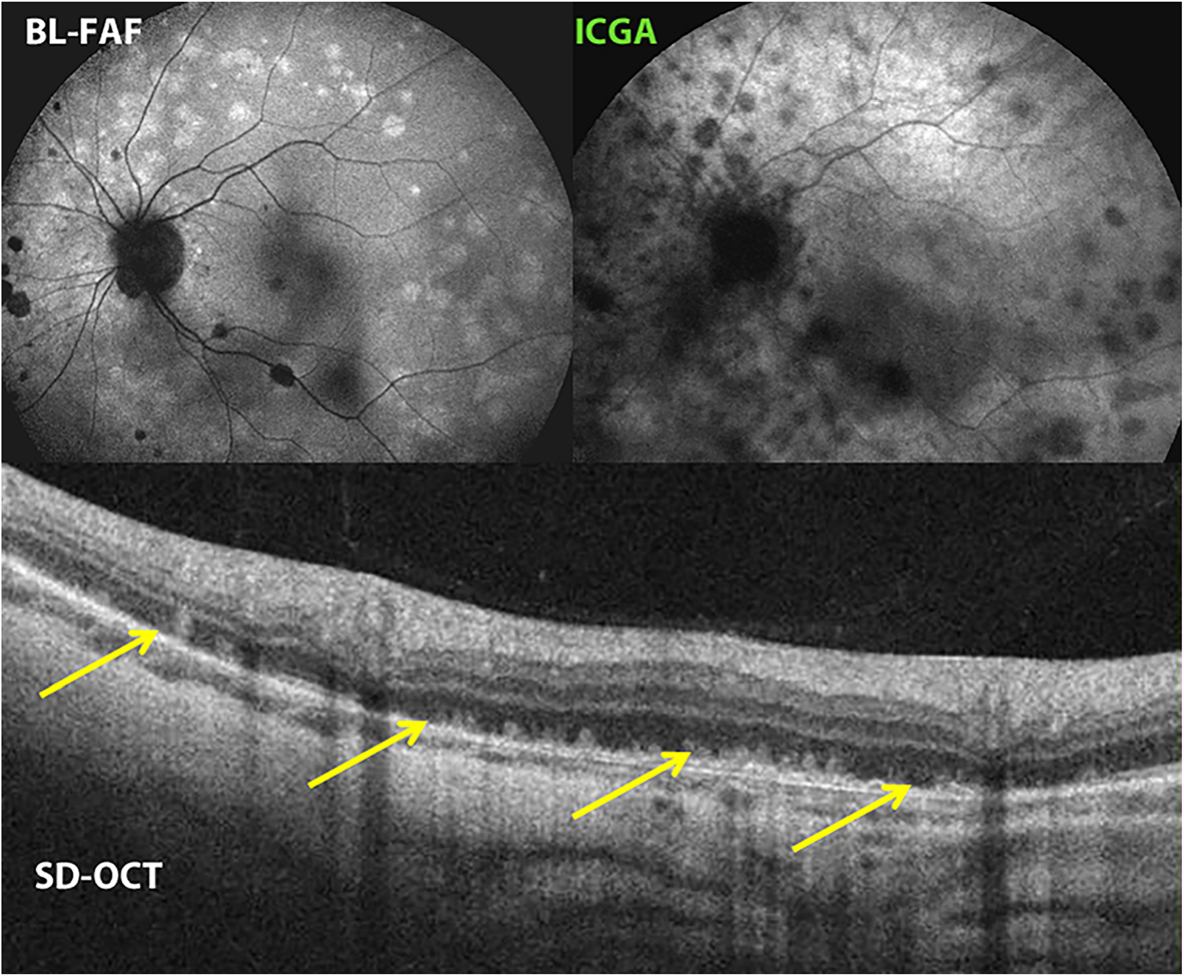
Multifocal choroiditis (MFC). BL-FAF, ICGA and SD-OCT. Loss and/or damage of the photoreceptor outer segments (bottom picture SD-OCT, yellow arrows), corresponding to the areas of BL-FAF-hyperautofluorescence (top left) and to the areas of ICGA hypofluorescence (top right)

Spectral domain optical coherence tomography (SD-OCT) is very helpful in MFC, like for all other PICCPs. In MFC, OCT pictures show that the degree of repercussion of choriocapillaris non-perfusion on the outer retina (and sometimes even inner retina) can be pronounced in the active phases of MFC when compared to the more benign choriocapillaritis entities such as MEWDS. It clearly shows damage and/or loss of the outer segments of photoreceptor cells co-localising with areas of hyperautofluorescence on BL-FAF and areas of hypofluorescence on ICGA (Fig. 5).

**Fig. 6:**
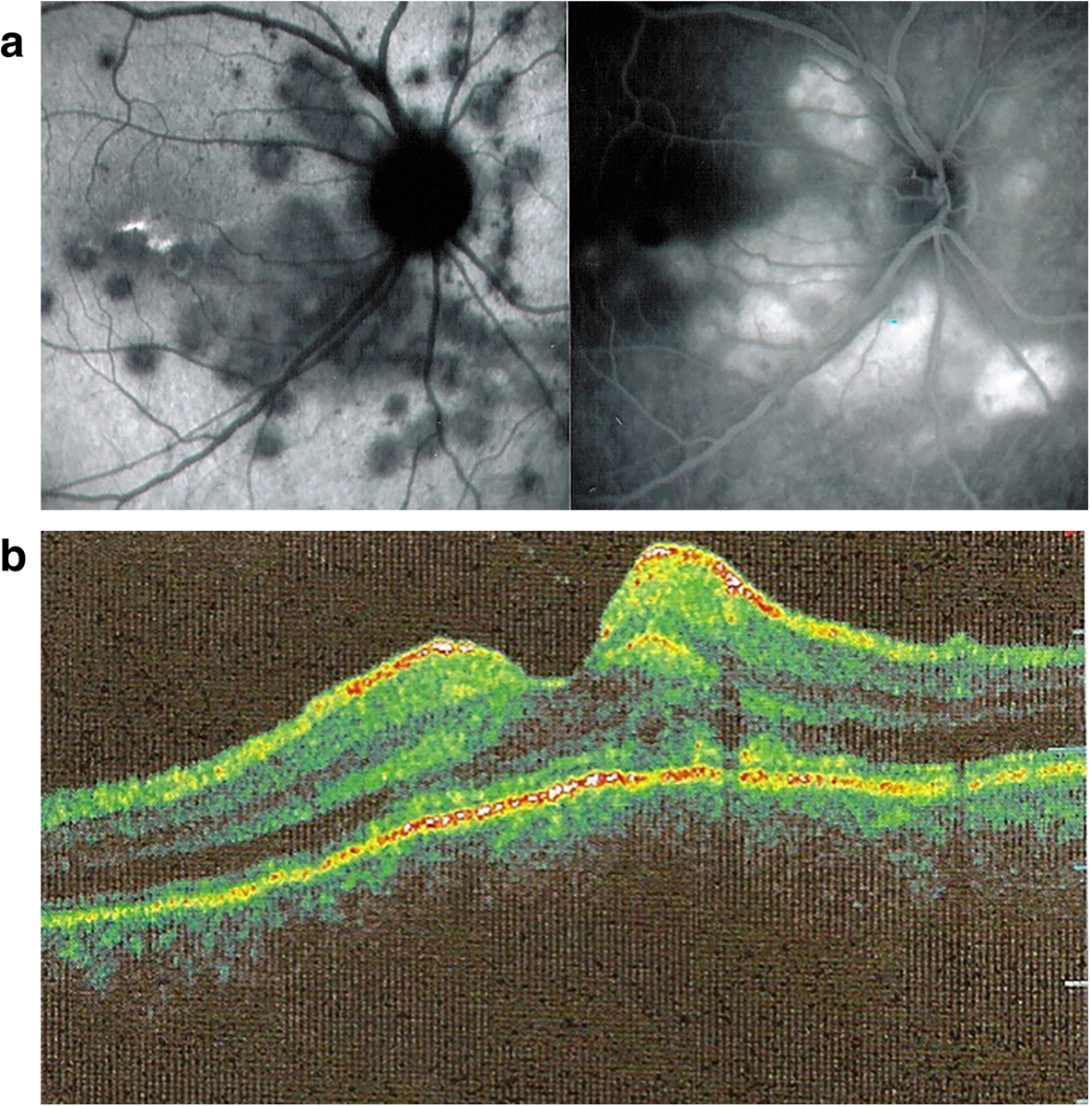
Multifocal choroiditis (MFC). ICGA & FA of acute episode. Areas of ICGA hypofluorescence around optic disc indicating severe choriocapillaris non-perfusion (left picture). On the right image, late FA frame showing diffuse hyperfluorescence due to pooling of dye in the retinal and subretinal space due to probable exudation from retinal vessels due to severe outer retinal ischaemia, as shown in another case by OCT on Fig. 6b. (Partially reprinted from Diagnostics (Basel). 2021 May 24;11 (6):939. 10.3390/diagnostics11060939.). b. Multifocal choroiditis (MFC) patient with outer retina ischaemia and photoreceptor outer segment damage. Intra and subretinal fluid probably coming from retinal reactive permeability increase and exudation induced by outer retinal ischaemia due to choriocapillaris non-perfusion

Similar to APMPPE/AMIC, the consequences of choriocapillaris nonperfusion can involve the whole thickness of the retina in the areas of choroiditis with pooling of fluid under the retina around the focus of chorioretinitis. This severe ischaemia might indicate that larger choroidal vessels seem to be involved as suggested by the only histopathological report available [11]. In a case of APMPPE/AMIC with similar features, the authors concluded that the retinal changes were due to ischaemia of the outer retina [53]. Severe outer retinal ischaemia can produce compensatory dilatation and permeability of the inner retinal vessels producing accumulation of intraretinal and subretinal fluid [54] (Fig. 6a & b).

OCT reports on MFC show disruption of the outer segments of the photoreceptors which correspond to hyperautofluorescent plaques on BL-FAF [55]. It is understandable that OCT findings in MFC and APMPPE/AMIC are similar as it is the same mechanism of choriocapillaris closure with more or less severe consequences on the retina.

### Optical coherence tomography angiography (OCT-A)

**Fig. 7:**
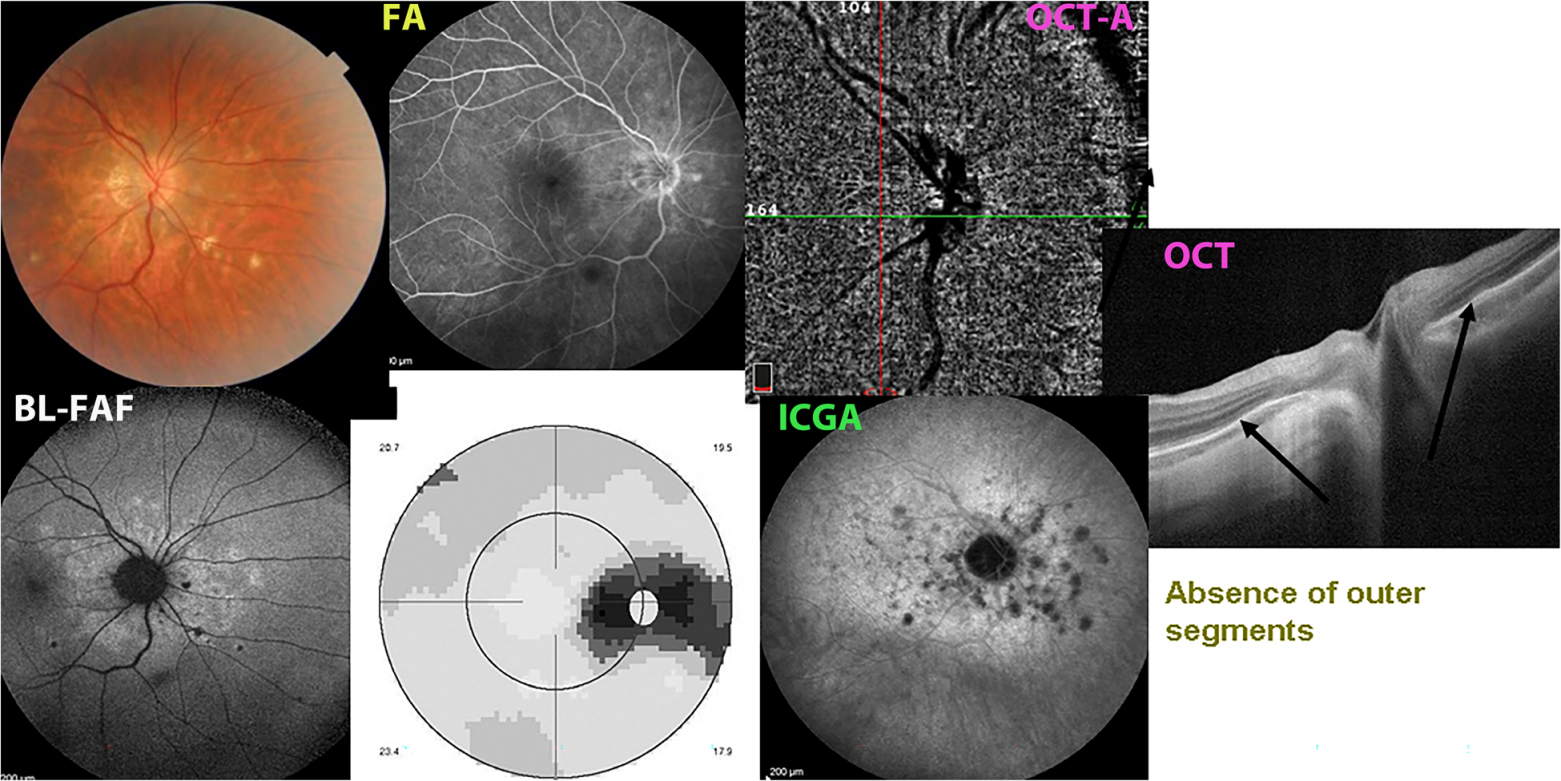
Case of Multifocal choroiditis (MFC). Comprehensive multimodal imaging. Multiple yellow foci in fundus typically seen in MFC (top left). Circum-papillary hyperautofluorescence on BL-FAF picture (bottom left) without FA signs (top middle) and blind spot enlargement on visual field (bottom middle). ICGA hypofluorescence clearly shows occult choriocapillaris non-perfusion, corresponding to BL-FAF hyperfluorescence. Lesions are barely seen on OCT-A (top right), while OCT shows loss or damage to photoreceptor outer segments (extreme right picture)

In contrast to MEWDS where small end-capillary vessels with low flow are not detected by OCT-A, in MFC OCT-A can show choriocapillaris drop-out, as the vessels involved are larger precapillary vessels. However, with presently used conventional OCT-A instruments, ICGA is still more precise (Fig. 7), while swept-source (SS)-OCT may represent a promising and useful tool for the upcoming future.

**Fig. 8:**
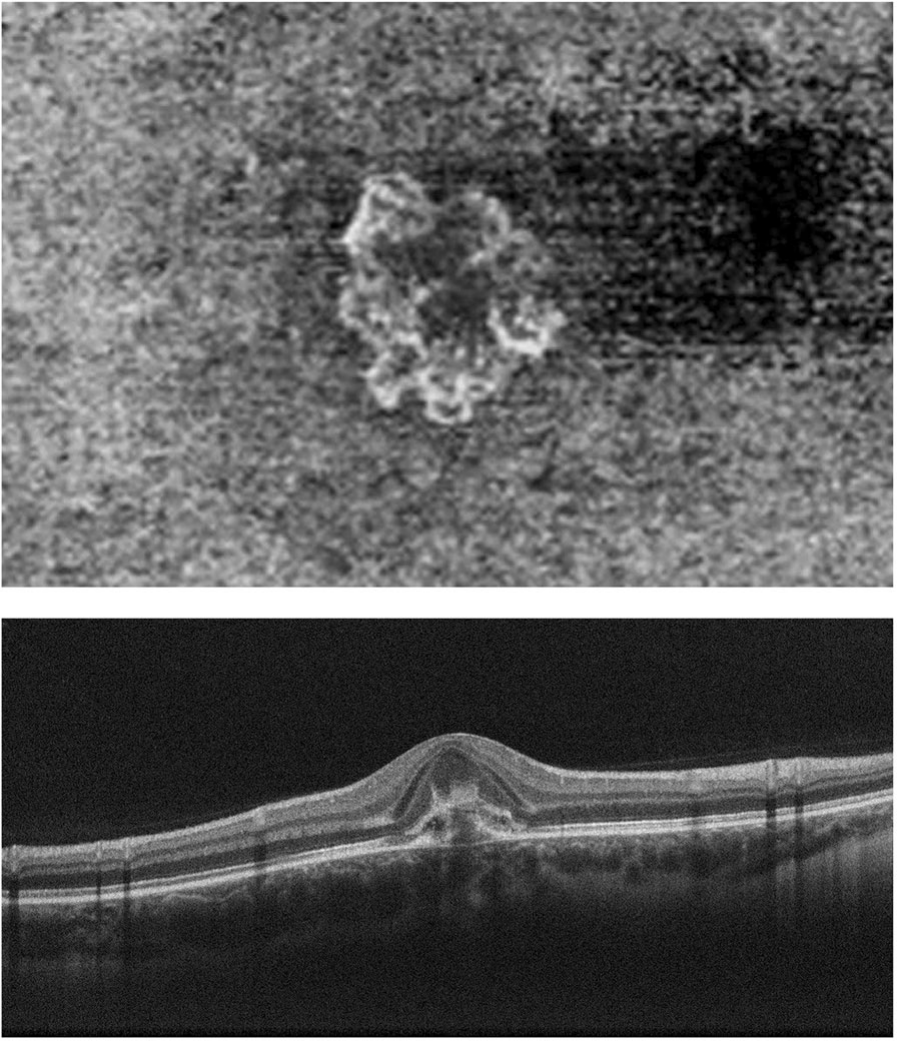
Multifocal choroiditis (MFC), OCT-A. Neovascular net clearly shown by en face OCT-A (top) and OCT.

Beside choriocapillaris drop-out, OCT-A is especially useful to detect CNVs and detect their evolution after anti-VEGF treatment [56] (Fig. 8).

### Fundus autofluorescence (FAF)

Blue-light fundus autofluorescence (BL-FAF) is a recent imaging modality based on the capacity of a scanning laser ophthalmoscope (HRA2, Heidelberg) to detect fluorophores within the RPE. In case of impaired cellular metabolism, lipofuscin accumulates within the RPE cells increasing the amount of autofluorescence. On the other hand, when the cells’ metabolism is completely arrested or in case of cell death there is no more emission autofluorescence [57, 58].

**Fig. 9:**
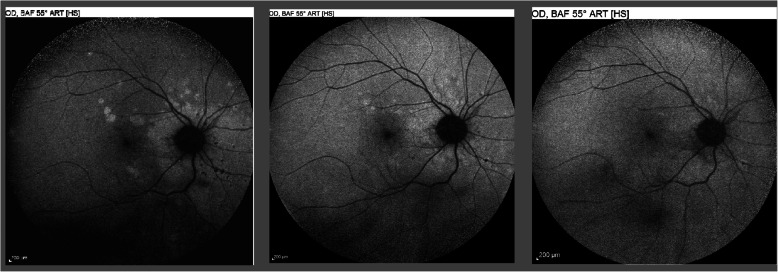
Multifocal choroiditis (MFC), BL-FAF. Hyperautofluorescence around optic disc at presentation (left picture) that decreases after introduction of corticosteroid/immunosuppressive therapy (middle picture) and disappears at the end of therapy (right picture)

Fundus autofluorescence in MFC shows increased autofluorescence in those areas that have silent (meaning absent FA signs), corresponding to ICGA hypofluorescent areas. The hyperautofluorescence is not explained by the usual mechanism but by the absence of the usual screen of photoreceptor outer segments allowing to better “see” the normal lipofuscin content of RPE cells **(**Fig. 9).

BL-FAF is hypoautofluorescent in the cicatricial areas. After corticosteroid / immunosuppressive therapy hyperautofluorescence of active disease areas disappears in parallel with resolution of ICGA hypofluorescence. The areas showing hyperautofluorescence go beyond the ICGA hypofluorescent areas indicating that dysfunction of cells and inflammatory involvement go even beyond the areas detected by ICGA. A recent study showed that BL- FAF allowed to detect, like ICGA, widespread occult inflammatory lesions in idiopathic CNVs, confirming the effect of inflammatory choriocapillaris non-perfusion on the outer retina, although this was not the interpretation by the authors of FAF signs of this report [59].

### Multimodal imaging is the key to understanding choriocapillaritis entities including MFC

**Fig. 10:**
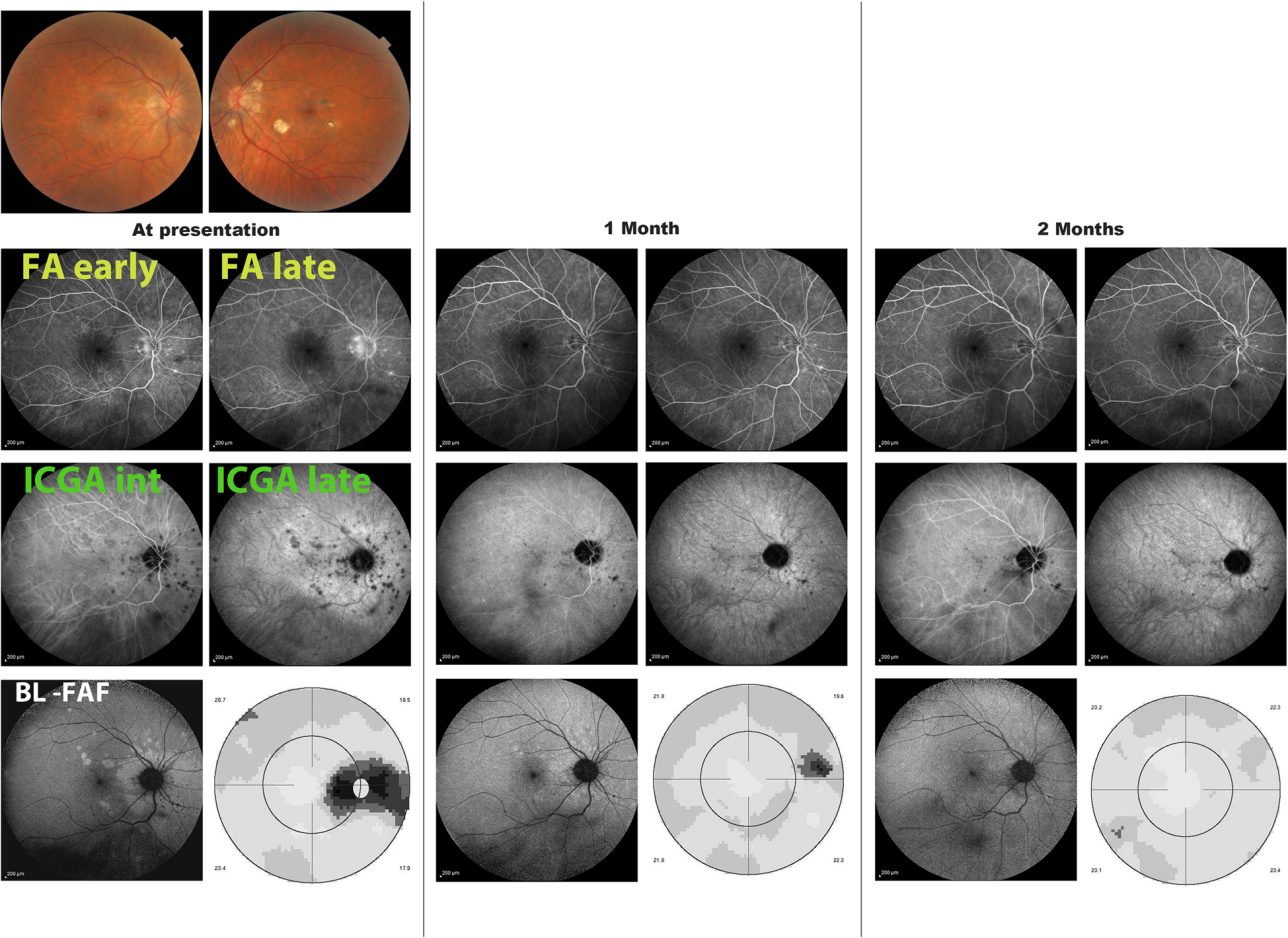
Multifocal choroiditis (MFC) Multimodal imaging monitoring of evolution of lesions following corticosteroid/immunosuppressive treatment. At presentation FA only shows old punctiform cicatricial hyperfluorescent pinpoints nasal to disc. ICGA (late frame) shows the numerous new lesions corresponding to BL-FAF hyperautofluorescence. After 2 months of treatment, BL-FAF, ICGA and visual field have recovered

**Fig. 11:**
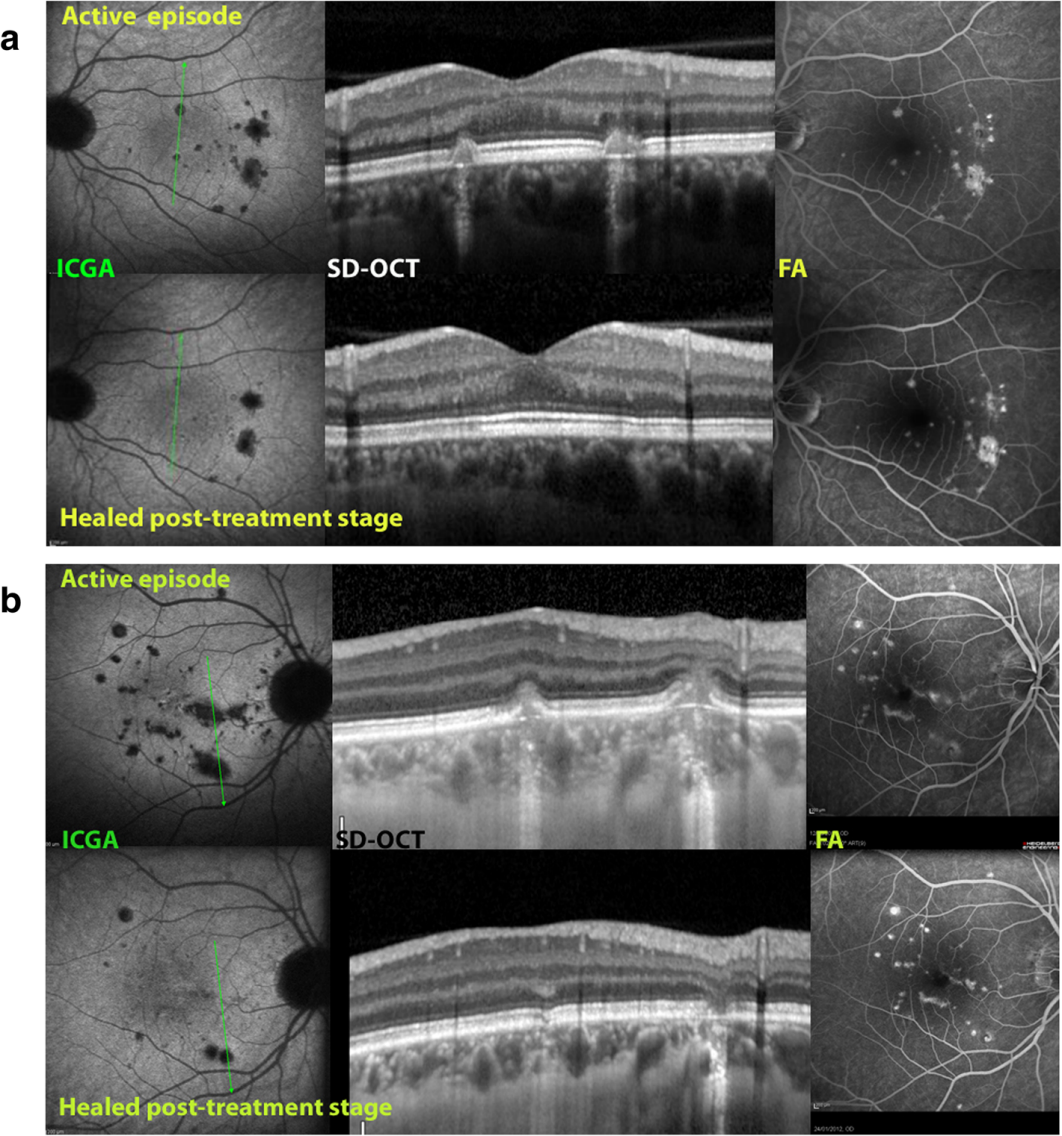
Case of MFC during acute episode (top 3 images) and after treatment (bottom 3 images. b. Case of MFC during acute episode (top 3 images) and after treatment (bottom 3 images. Figures 11a & b: Spectralis - ICGA/FA/ and OCT images in two cases of MFC. During the active phase of the disease (top lines of pictures), OCT findings disclose the presence of two inflammatory lesions showing damaged and clumped photoreceptor outer segments; RPE layer appears interrupted at its peak with, in the second case (11b), material infiltrating the subretinal space without the involvement of the inner retina. In the quiescent phase (bottom lines of pictures), OCT shows the resolution of the agglomerated material. It is also interesting to note that the FA images (right frames) do not permit to distinguish the active phase versus the quiescent phase of the disease

Multimodal imaging has contributed essentially to the understanding of PICCPs such as MFC. However, the term multimodal is not having the same signification from one article to another. Unfortunately, in many such ”to multimodal imaging” reports, ICGA is not included, albeit it is crucial in the imaging appraisal choriocapillaritis diseases [60] (Figs. 7, 8, 9, 10 & 11).

### Visual field testing

**Fig. 12:**
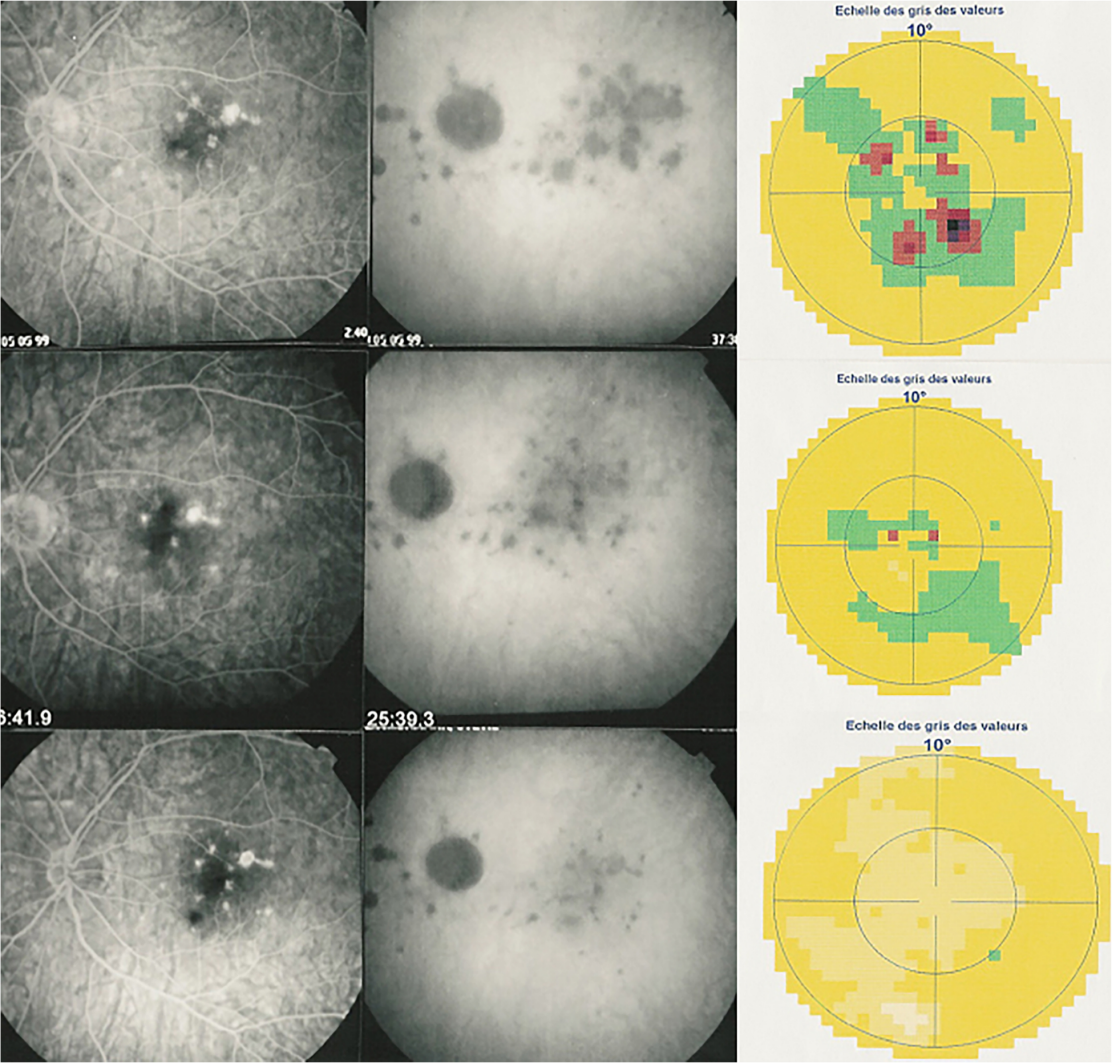
Correlation between FA, ICGA and visual field during corticosteroid treatment of MFC. This myopic patient presents with left eye photopsias, left decrease of visual acuity (VA) and the presence of a subjective left scotoma. FA (top left) only shows cicatricial FA lesions corresponding to cicatricial fundus lesions seen on fundus examination. ICGA (top middle) shows widespread hypofluorescent areas indicating fresh choriocapillaris lesions with corresponding visual field defects (top right). During corticosteroid therapy, practically no change is seen on FA, whereas ICGA signs of active disease progressively resolve together with visual field improvement (right column). (Reprinted from Diagnostics (Basel). 2021 May 24;11 (6):939. 10.3390/diagnostics11060939)

Visual field testing can show small scotomas corresponding to chorioretinal scars. In the active phase however, scotomas are larger and correspond to choriocapillaris non perfusion shown on ICGA. Visual field recovery is well correlated with the regression of ICGA hypofluorescent areas that occurs following sub-Tenon’s or systemic corticosteroid therapy (Fig. 12). On the other hand, recovery is not well correlated to FA.

As for all PICCPs, enlargement of the blind spot can be seen in MFC and is explained by peripapillary choriocapillaris non-perfusion and consecutive damage to the photoreceptor outer segments [33, 51].

## Diagnosis and differential diagnosis

As for all PICCPs an infectious cause should be excluded as a first step. Many infectious causes can produce a clinical picture resembling MFC, including tuberculous choroiditis, West-Nile virus choroiditis, Candida choroiditis, bacterial emboli and more rare diseases such as pneumocystis choroiditis and choroidal coccidiomycosis. Among the non-infectious entities, ocular sarcoidosis has to be excluded.

Finally, when evidence for histoplasma capsulatum infection is found the diagnosis of Histoplasma positive “Presumed Ocular Histoplasmosis Syndrome” (POHS) can be made.

## Treatment

Reports on management of MFC are very scarce. Most reports concern the treatment of MFC associated CNVs. There was, however, anecdotal evidence to favour corticosteroid therapy that in most cases should be associated to non-steroidal immunosuppressive therapy in case of newly diagnosed active disease or reactivation of multifocal choroiditis [38, 61].

A recent well conducted study including 32 patients confirmed that immunosuppressive therapy significantly decreased not only the number of recurrences of MFC but also the number of anti-VEGF injections in the cases with CNVs [62]. Monitoring of therapy is best done by ICGA which is equally or more sensitive than visual field testing to detect actively involved areas and response to therapy showing regression of hypofluorescent areas. BL - FAF is equally useful as ICGA to follow and monitor the regression of new active lesions and has the advantage to be non-invasive. It was also shown that after corticosteroid treatment recovery, choroidal blood flow velocity increased, and choroidal thickness decreased indicating that the choroid is involved beyond the choriocapillaris [63].

**Fig. 13:**
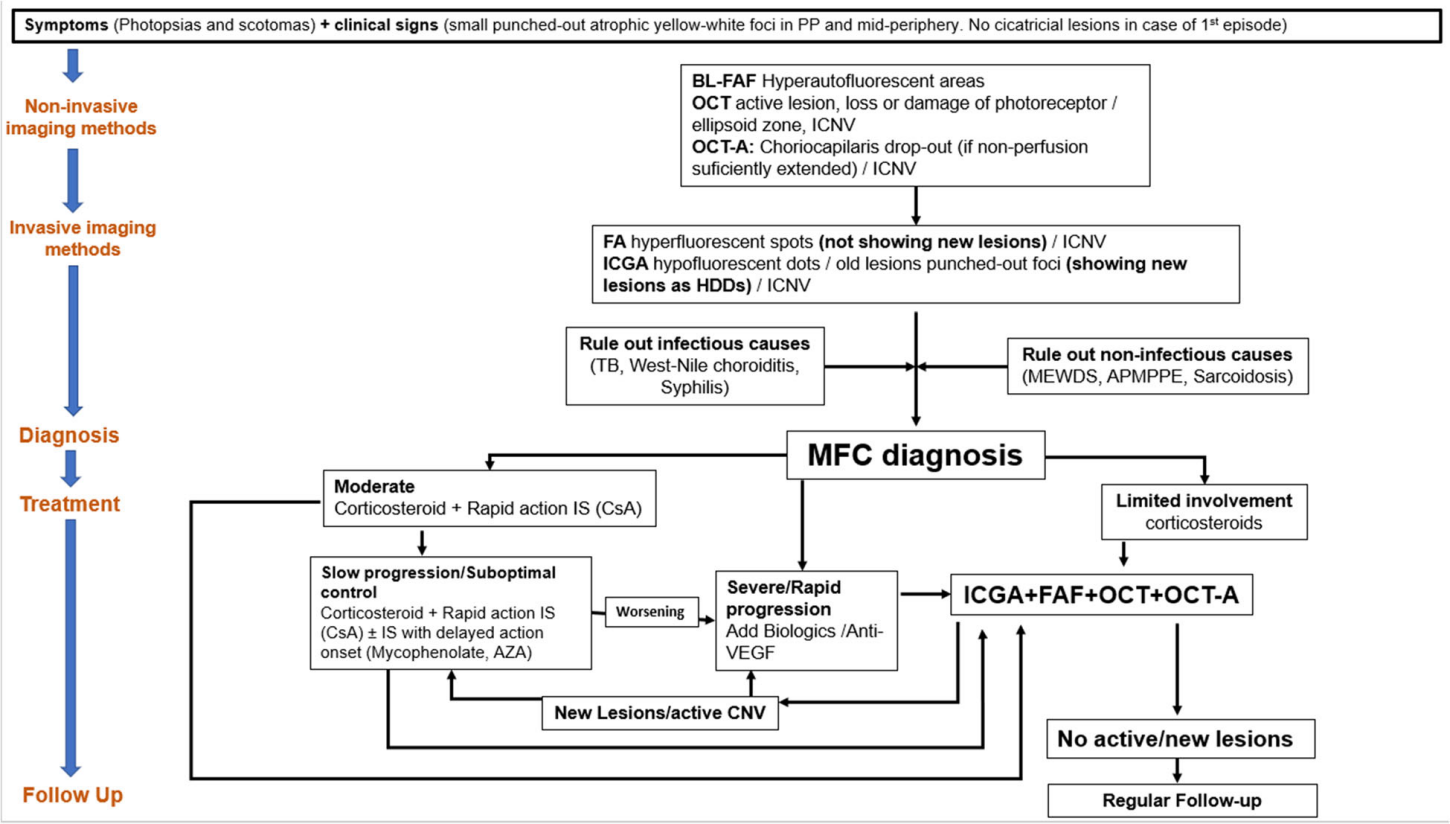
MFC: Diagram of Imaging appraisal in practice; diagnosis and management following the logical sequence of exams. PP: posterior pole. OCT: optical coherence tomography. iCNV: inflammatory choroidal neovascularization. BL-FAF: blue light autofluorescence. OCT-A: OCT-angiography. FA: fluorescence angiography. ICGA: indocyanine green angiography. HDDs: hypofluorescent dark dots. TB: tuberculosis. MEWDS: multiple evanescent white dots syndrome. APMPPE: acute posterior multifocal placoid pigment epitheliopathy. MFC: idiopathic multifocal choroiditis. IS: immunosuppressors. CsA: Cyclosporine. AZA: Azathioprine

Corticosteroids can be given initially by sub-Tenon’s injections if the reactivation is unilateral but have to be given systemically if there is no response or if the involvement is bilateral. For rapid action, corticosteroids are recommended. In most cases they are however not sufficient and bear with them deleterious side-effects. In severe cases Immunosuppressive agents should be added. We usually use cyclosporine (2.5–4.5 mg/kg) or tacrolimus (0.05–0.2 mg/kg), rapidly acting agents, together with Mycophenolate mofetil 1.5–3 g, a cytostatic agent that needs several weeks to reach efficient action. Azathioprine (2–2.5 mg/kg) has a similar mode of action as mycophenolate but is slightly less well tolerated. Biologic agents can be used in cases insufficiently well controlled by classical immunosuppression (Fig. 13). In case of MFC complicated by CNVs, the approach has to be more aggressive by combining systemic steroids and systemic immunosuppressive with intravitreal injection of anti-VEGF agents. The well-known role of immunosuppressive agents in the treatment of inflammatory CNVs was confirmed by Neri et al. [64]. A prompt and appropriate use of systemic immunosuppression associated with steroids and anti-VEGF agents leads to better visual outcome as well as long term control of CNV activity compared to steroids and anti-VEGF only [65].

## Case series

### Aim

As mentioned before, MFC belongs to the more severe end of the spectrum of PICCPs, and treatment should be appropriate and sustained. The purpose of our study was to demonstrate the benefit of aggressive multiple immunosuppressive therapy, in order to stop the deleterious course of the disease.

### Patients and methods

This retrospective case series was performed in the Centre for Ophthalmic Specialised care (COS), Lausanne, Switzerland. Patients diagnosed from 1994 to 2020 with idiopathic multifocal choroiditis (MFC) treated with multiple immunosuppressants were included. Patients not treated with multiple immunosuppressants or with insufficient follow up were the exclusion criteria. Imaging analysis included spectral domain optical coherence tomography (SD-OCT) and enhanced depth imaging OCT (EDI-OCT) (Heidelberg Engineering GmbH, Heidelberg, Germany), OCT angiography (OCT-A) (AngioVue®, Optovue, Fremont, CA, USA) Fluorescein and Indocyanine angiography (FA, ICGA) (Heidelberg Engineering GmbH, Heidelberg, Germany) before and after the instauration of the treatment. Best corrected visual acuity (BCVA), intraocular pressure (IOP) and routine ocular examination, as well as laser flare photometry (LFP) were performed at presentation and during the follow up of patients. Immunosuppression comprised at minimum two among the following agents: prednisone, cyclosporine, azathioprine, mycophenolic acid and infliximab. Mean duration of therapy was calculated.

### Results

From 1994 to 2020, 26 of 2102 new patients (1.24%) were diagnosed with MFC. Most patients were sent for a second opinion. 25 (96%) patients were women and only 1 (4%) was man. Mean age was 35.3 ± 12.7 years. Among the 52 eyes, 43/52 (82%) were myopic with a mean dioptre value of − 5.87 ± 2.94, six (12%) eyes were hypermetropic with mean dioptre value of 2.0 ± 2.68 and 3 (6%) were emmetropic. 14/52 (27%) eyes had at least 1 anti-VEGF injection because of choroidal neovascularisation (CNVs), 1 eye had a phototherapy laser treatment and 37/52 (71%) had no complication of CNVs during the follow-up. 5/26 (19%) patients had a long-term follow-up and fulfilled the inclusion criteria, having been treated with at least two immunosuppressants with sequential monitoring. Mean age was 26.4 ± 9.3 years. Snellen best corrected visual acuity (BCVA) at presentation was 0.955 ± 0.26 and 0.9 ± 0.24. Mean follow up was 84 ± 55 months and mean treatment duration was 52 ± 18 months. LFP at presentation was 6.34 ± 2.94 ph/ms and at last follow-up it was 6.2 ± 3 ph/ms. Visual field mean defect was 4.64 ± 3.1 at presentation and 3.9 ± 1.9 at last follow-up. Treatment with multiple immunosuppressive agents was shown to stop the progression of the disease in the 4 cases with prolonged follow-up and prolonged treatment. In the last patient follow-up and treatment were less than 4 months and there was still activity of the CNV but no occult active disease areas on ICGA and BL-FAF.

### Summary of findings

Data on patients are summarised on Table 3.

**Table 3:** Patients, treatment data, visual acuities, visual fields and follow-up

Name	Age	Refraction	Activity now	VA 1	VA last	Flare 1 (ph/ms)	Flare 2	VF MD first	VF MD last	Month of f-up	Pred/ne	my/late	CsA	CNV
Pat 1 OD	14 Y	−2.25	no	0,6	0,5	9,4	10,5	10,8	5,5	84	50 mg	2 g	250 mg	no
Pat 1 OS		−2.00	no	1,25	1,25	3,4	3,9	3,1	0,7		50 mg	2 g	250 mg	no
Pat 2 OD	26 Y	−7,75	no	1,00	1,00	5	5,3	4,6	5,2	60	50 mg	0	200 mg	no
Pat 2 OS		−8.00	no	1,25	1,25	4,6	3,8	1	0,9		50 mg	0	200 mg	no
Pat 3 OD	40 Y	−7,25	no	0,9	0,8	9,2	11,1	2,5	5,3	132	50 mg	720 mg	150 mg	no
Pat 3 OS		−6,25	no	0,8	1,00	12,4	6,6	8,6	2,9		50 mg	720 mg	150 mg	no
Pat 4 OD	26 Y	−11.00	no	1,00	1,00	5,4	9,3	1,3	5,3	140	50 mg	1440 mg	200 mg	no
Pat 4 OS		−12.25	no	1,00	1,00	4	4,1	5,8	5,4		50 mg	1440 mg	200 mg	no
Pat 5 OD	24 Y	−8.25	no	1,00	0.8	4,7	3.2	4,5	2.8	4	40 mg	1440 mg	400 mg	no
Pat 5 OS		− 8.25	yes	0,5	0.5	5,3	4.2	4,2	5		40 mg	1440 mg	400 mg	yes

*Pat* Patient, *1* at presentation, *2* at last follow-up

*OD* Oculus dexter, *F-up* follow up

*OS* Oculus Sinister

*Y* years old, *my/late* mycophenolate

*VA* Visual acuity, *CsA* cyclosporine

*VF* Visual field, *CNV* choroidal neovascularisation

*MD* mean defect, *ph/ms* photons par millisecond, *mg* milligram, *g* gram

## Conclusion

Idiopathic multifocal choroiditis is part of the group of primary inflammatory choriocapillaropathies (PICCPs). It groups the different entities that were considered separately such as PIC or POHS in non-endemic areas with negative search for Histoplasma capsulatum. It is on the more aggressive side of the spectrum of PICCPs and most of the time needs systemic corticosteroids associated with non-steroidal immunosuppression.

## Abbreviations

MFC: Idiopathic multifocal choroiditis
PICCPs: Primary Inflammatory Choriocapillaropathies
SD-OCT: Spectral domain optical coherence tomography
EDI-OCT: Enhanced depth imaging OCT
OCT-A: OCT angiography
FA: Fluorescein angiography
ICGA: Indocyanine angiography
BCVA: Best corrected visual acuity
IOP: Intraocular pressure
LFP: Laser flare photometry
CNVs: Choroidal neovascularisation
MEWDS: Multiple evanescent white dot syndrome
APMPPE: Acute posterior multifocal placoid pigment epitheliopathy
AMIC: Acute multifocal choriocapillaritis
PIC: Punctate inner choroidopathy
POHS: Presumed ocular histoplasmosis syndrome
BL-FAF: Blue-light fundus autofluorescence
anti-VEGF: Anti-vascular endothelial growth factor
SS-OCT: Swept-source-OCT
